# Novel Presentation of Parechovirus Encephalitis in Children: Two Unique Cases

**DOI:** 10.7759/cureus.26456

**Published:** 2022-06-30

**Authors:** Trupti Pandit, Ramesh Pandit, Lokesh Goyal, Kunal Ajmera, Naresh Dasari

**Affiliations:** 1 Pediatrics/Hospital Medicine, Inspira Medical Center, Vineland, USA; 2 Hospital Medicine, University of Pennsylvania Health System (Penn Medicine) / Chester County Hospital, Philadelphia, USA; 3 Hospital Medicine, CHRISTUS Spohn Hospital, Corpus Christi - Shoreline, Corpus Christi, USA; 4 Epidemiology, George Washington University, Washington D.C., USA; 5 Internal Medicine, Roger Williams Medical Center, Providence, USA

**Keywords:** pediatric infectious disease, pediatric hospital medicine, acute encephalitis, post-infectious, viral illness, altered mental status, movement disorder, dystonia, encephalitis, parechovirus infection

## Abstract

Human Parechovirus encephalitis is an uncommon infection. Very few of the cases have been reported in the literature so far. These reports are mainly about neonatal encephalitis, primarily affecting preterm neonates. Parechovirus encephalitis in otherwise healthy pediatric populations is a rare entity. Here, we present two unique pediatric cases secondary to Parechovirus infection, one with hemidystonia and another with acute onset of altered mental status, confusion, and headache.

## Introduction

Human Parechovirus (HPeV) has at least 16 subtypes, which can cause infections like febrile illness, respiratory illness, and neurological diseases [1]. HPeV1 is a widely spread pathogen that affects mainly young children, commonly associated with mild gastrointestinal or respiratory symptoms. HPeV2 infections are primarily associated with gastrointestinal symptoms. HPeV3 has been associated with more severe diseases, such as neonatal sepsis and meningitis [2]. Despite the common prevalence of the HPeV infection in young children, Parechovirus encephalitis is rare in healthy pediatric populations [3]. Here, we present two interesting cases of human Parechovirus encephalitis with differing presentations.

## Case presentation

Case 1

The patient is a five-year-old African American male with a past medical history significant for reactive airway disease and failure to thrive, who presented with fever, which started 10 days before presentation to our hospital. The maximum temperature was reported at 103°F. After two days, he developed a widespread vesicular rash, including hands and feet but not involving his mouth. He was diagnosed with an ear infection and was started with a 10-day course of amoxicillin by his pediatrician as an outpatient. Three days before the presentation, he was noticed to hold his left hand unusually, flexing his fingers, wrist, and elbow, sometimes also turning his shoulder in abduction. He would not open his left hand unless asked to do it. The next day, his mother noticed that he was limping and toe-stepping with his left foot. His left foot was also rotated internally. The same day, his teacher noticed that he could not study as usual. He could not cut well with scissors and almost fell on the playground. The patient's rash was not completely cleared, but it had improved. He presented to the hospital with a left-sided weakness for three days. The patient had otherwise been talking and acting like himself. He did not complain of any pain and denied headaches, speech problems, or vomiting.

Past medical history included birth at 37 weeks of gestation, with no prenatal complications. Per his mother, he is a very picky eater. On presentation, his vitals were as follows: temperature 36.7°C (98.1°F), respiratory rate 32/min, blood pressure 91/63 mmHg, pulse 84/min, and weight 16.2 kg (seventh percentile for age).

He was alert, awake, and interactive with the examiner during the examination. His heart and lung examinations were normal. He was noted to have a resolving skin rash over his hands and legs with hyper-pigmented lesions. His muscle tone was increased on the left side compared to the right. His strength was 5/5 on the right side but 4+/5 on the left upper and lower extremities. The patient was holding his hand in a bird beak kind of posture with the wrist and the metacarpophalangeal joints partially flexed. Proximal interphalangeal and distal interphalangeal joints were held in extended positions. He could open his palm when asked to do so, but it returned to the above-described posture when distracted. He was holding his left foot in a plantar-flexed, inverted position and walking on tiptoes. His reflexes were 1 + bilaterally. His sensations were normal bilaterally. The cranial nerve examination was within normal limits. Finger to nose and the heel-to-shin tests were normal on the right side but difficult to perform on the left side.

His investigations showed normal complete blood count (CBC), basic metabolic panel (BMP), and computed tomography (CT) of the brain. Cerebrospinal fluid (CSF) analysis results included white blood cells (WBC) 2/microL, glucose 58 mg/dL, and protein 18 mg/dL. The Parechovirus nucleic acid amplification test (NAAT) was positive. Enterovirus, herpes simplex virus ( HSV), arbovirus, brucella, Bartonella, human immunodeficiency virus (HIV), tick-borne disease, Lyme, mycoplasma, and West Nile virus tests were negative. The venereal disease research laboratory (VDRL) test was negative and an antinuclear antibody test (ANA) was borderline high to the 40s. The double-stranded deoxyribonucleic acid (Ds-DNA ) test was negative. The immunoglobulin panel was normal. The magnetic resonance imaging (MRI) of the brain showed a long repetition time (TR) with hyperintense lesions in the globus pallidus, the right larger than the left. A patchy enhancement was also seen in the right globus pallidus. Another long TR hyperintense lesion was seen with avid postcontrast enhancement and restricted diffusion in the right cerebral peduncle. The MRI of the cervical spine was negative.

This is more consistent with an inflammatory response, but given the clinical course of a previous infection, this presentation is likely postinfectious etiology. The presence of amplifiable ribonucleic acid (RNA) of Parechovirus in CSF was a relative contraindication to the use of corticosteroids or other immunosuppressants. 

The patient underwent physiotherapy, which did not provide adequate relief. He required muscle relaxants, including Baclofen and Klonopin, with partial improvement in the dystonia from medications. Repeat MRI after one month suggested stable lesions.

Case 2

An eight-year-old Caucasian female patient without a significant past medical history presented with altered mental status. On the day of the presentation, she was normal in the morning, and around mid-day, she started with an acute onset of blurry vision followed by a left frontal headache. Following this, she developed difficulty speaking. Per her mother, she was incoherent with her speech and was "wobbly on her feet." She has six episodes of emesis en route to the hospital via emergency medical services (EMS). She continued to have difficulty speaking and answering questions appropriately in the hospital. She also had brief periods of apnea, as well as continued headaches, nausea, and vomiting. Her mental status was waxing and waning intermittently. The patient's history included being born at 40 weeks of pregnancy without any pre or postnatal complications. There was reported diarrheal illness a few weeks prior to symptom onset, with similar symptoms among family members a day prior, but no flu-like symptoms, cough, fever, or rash were noted prior to presentation. Denies history of migraine with or without aura, or seizure disorder. The mother denied the use of any over-the-counter or herbal medications. Family history was significant to have brother and mother having viral gastroenteritis a day earlier. There was no family history of seizures.

On presentation, her vitals were as follows: temperature 36.8°C (98.2°F), blood pressure 109/56 mmHg, pulse 100/min, respiratory rate of 28/min, weight of 27 kg, and oxygen saturation of 100% on room air. Upon physical examination, she was drowsy but was able to open her eyes on verbal commands. The head was normocephalic and atraumatic, and heart, lung, and abdomen examinations were unremarkable. Pupils were 4 mm, round, and reactive to light. During the neurological exam, she appeared lethargic and had delayed responses to orientation questions. The cranial nerve examination was unremarkable, and the motor examination showed good muscle tone and 2+ reflexes bilaterally. Sensations were intact, and no lack of coordination was noted. Physical examination maneuvers for nuchal rigidity, Kernig's and Brudzinski's signs, were negative and no skin rash was noticed.

The CT scan of the head was unremarkable. The patient also had negative urine and blood drug screen tests. White blood cell (WBC) count was elevated to 12.7 K/ul. Initial glucose was 147. CSF studies show a WBC count of 85, red blood cell (RBC) at 730, with normal protein and glucose levels. She received vancomycin and ceftriaxone empirically in the emergency department. CSF culture remained negative for bacterial infections, and antibiotics were discontinued. Viral testing for Parechovirus NAAT came back positive with negative Enterovirus NAAT. The patient remained hemodynamically stable, and her mental status improved over the next two days. The patient was discharged home in stable condition after supportive care. Table 1 below shows the comparison between the two case presentations.

**Table 1:** Presentations and key findings of cases 1 and 2 T: temperature; RR: respiratory rate; BP: blood pressure; HR: heart rate; SpO_2_: oxygen saturation; CBC: complete blood count; BMP: basic metabolic panel; TR: repetition time; NAAT: nucleic acid amplification test; CSF: cerebrospinal fluid

Demographic	Case 1	Case 2
Age (year)	5	8
Race	African American	Caucasian
Gender	Male	Female
Symptoms	Fever, rash after 10 days: left-sided weakness, dystonia, limping,	Blurry vision, headache, speech difficulty, vomiting, and altered mental status
Duration to onset of neurological symptoms	10 day	1 day
Vitals	T: 36.7°C, RR: 32/min. BP: 91/63mmHg, HR: 84/min, SpO_2_: 100% on room air (RA), weight: 16.2 kg	T: 36.8°C, HR: 100/min, RR: 28/min, BP: 109/56 mmHg, SpO_2_: 100% on RA, Weight: 27 kg
Lab workup	Normal CBC, BMP	WBC was elevated to 12.7K/ul. Serum glucose 147. Drug screens: negative
CSF	WBC: 2, glucose: 58, and protein: 18	WBC: 85, RBC: 730, protein and glucose levels within normal range
Parechovirus NAAT	Positive	Positive
Imaging study	MRI imaging: TR hyperintense lesions in the globus pallidus, the right > left. Patchy enhancement in right globus pallidus. Postcontrast enhancement and restricted diffusion in the right cerebral peduncle.	Normal CT scan

## Discussion

Parechovirus belongs to the family of picornavirus, a single-stranded, non-enveloped RNA virus with infections commonly seen in neonates. In our cases, the patients presented at a much later age. Parechovirus has at least 16 identified subtypes. The first Parechoviruses (E22 and E23) were isolated in 1956 and recognized as a new genus in 1996 as sequence analysis revealed them to be distinct from other picornaviruses. New Human Parechoviruses (HPeV) types 3-6 were recently identified. HPeV1 is known to affect mainly young children. HPeV1 infections are most commonly associated with mild gastrointestinal or respiratory symptoms, while HPeV2 infections are associated chiefly with gastrointestinal symptoms. HPeV3 has been associated with more severe diseases, such as neonatal sepsis and meningitis [2].

Usually, Parechovirus HPeV3 encephalitis is seen in neonates, especially premature ones. Neonates are likely, due to their immune status carry a guarded prognosis due to its impact on the neurodevelopmental outcome [4-6]. Key features of Neuroimaging include MRI T2 hyperintensity and corresponding diffusion restriction involving the periventricular and subcortical white matter, corpus callosum, and thalami, and most often symmetric [4]. In older kids, Parechovirus encephalitis is rarely reported in the literature and may raise the possibility of underlying immunodeficiency [7,8].

According to a study in Neonatal leucoencephalitis, HPeV is found in white matter, with white matter changes involving the periventricular and subcortical white matter [9]. These changes have been found more frequently in the frontal white matter, corpus callosum, internal capsule, external capsule, and the pyramidal tracts of the supratentorial brain and cerebral peduncle. Sparing of occipital white matter, thalamus, basal ganglia, and infratentorial regions were noted. The key differentiation from hypoxic ischemic encephalopathy (HIE) is that basal ganglia findings seem more frequent in HIE and the eventual disappearance of these lesions with Parechovirus infection.

Our cases differ from reported HPeV encephalitis as the first case presented with dystonia and the second presented with gastrointestinal symptoms followed by altered mental status. MRI brain findings also differ from prior reported cases as our patient’s MRI showed long TR with hyperintense lesions in the globus pallidus, the right larger than the left. Patchy enhancement is also seen in the right globus pallidus and restricted diffusion in the right cerebral peduncle.

## Conclusions

Parechovirus encephalitis is usually seen in the neonatal population but is rare in the healthy pediatric population. With these two unique pediatric cases having febrile illnesses with neurological symptoms including weakness, dystonia, and altered mental status, we wanted to add more information to the medical literature about HPeV encephalitis and various presentations and outcomes in the older pediatric population. Early detection and treatment are needed to minimize complications. We recommend keeping HPeVas a differential when managing patients with similar presentations. 
